# Is global health security worth 0.01% of our gross domestic product?

**DOI:** 10.1371/journal.pgph.0004491

**Published:** 2025-05-14

**Authors:** Gorik Ooms, Yibeltal Assefa, Salome Charalambous, Ter Tiero Elias Dah, Kristof Decoster, Bouke de Jong, Bernadette Hensen, Ryuichi Komatsu, Natama Hamtandi Magloire, Ellen M.H. Mitchell, Thijs Reyniers, Anna Rosanas-Urgell, Maria-Belen Tarrafeta-Sayas, Halidou Tinto, Alan Whiteside, Raffaella Ravinetto

**Affiliations:** 1 Department of Public Health, Institute of Tropical Medicine, Antwerp, Belgium; 2 Department of Public Health and Primary Care, Faculty of Medicine and Health Sciences, Ghent University, Ghent, Belgium; 3 School of Public Health, The University of Queensland, Brisbane, Australia; 4 The Aurum Institute, Johannesburg, South Africa; 5 University Ledea Bernard Ouedraogo of Ouahigouya, Ouahigouya, Burkina Faso; 6 Department of Biomedical Sciences, Institute of Tropical Medicine, Antwerp, Belgium; 7 Interfaculty Initiative in Planetary Health, School of Tropical Medicine and Global Health, Nagasaki University, Nagasaki, Japan; 8 Clinical Research Unit of Nanoro, Institut de Recherche en Sciences de la Santé, Nanoro, Burkina Faso; 9 Balsillie School of International Affairs, Waterloo, Canada; 10 University of KwaZulu-Natal, Durban, South Africa; 11 School of Public Health, University of the Western Cape, Cape Town, South Africa; PLOS: Public Library of Science, UNITED STATES OF AMERICA

## Introduction

Within days of starting his second term as President of the United States of America (US), Donald Trump ended most US contributions to global health. Global responses to HIV, tuberculosis (TB) and malaria are not the only programmes affected, but were particularly dependent on US support [1]. The US withdrawal from global health could result in 3 million additional HIV deaths and 10 million additional HIV infections; 107.000 additional malaria deaths and 15 million additional malaria infections; and 2 million of additional TB deaths, all in 2025 [2]. These decisions will negatively affect public health in the US and other countries that did not benefit from US aid. HIV, TB and malaria are global health security threats that require international collective action [3]. Undermining such collective action makes the world less safe for everyone.

At the time of writing, the Global Fund to fight AIDS, TB and Malaria (Global Fund) entered its replenishment cycle for 2027–2029, with a target of USD 18 billion. A failure of this replenishment would make it impossible for many countries to compensate for the absence of US funding *and* decreasing Global Fund support. As global health researchers, we call upon the international community to protect the global responses to HIV, TB and malaria. Beyond being *morally* the right thing to do, it would also be *pragmatically* smart, serving humanity’s collective interests.

## HIV, TB and malaria remain global health security threats

The abrupt end of most US funding for global health comes at a crucial moment for the fight against the three epidemics.

For *HIV*, the US President’s Emergency Plan for AIDS Relief (PEPFAR) contributed to scaling up prevention and treatment, increasing antiretroviral treatment (ART) coverage from about 1 million (2000) to 30 million (2024) [4]. The availability and scale-up of new technologies, particularly long-acting injectable antiretrovirals (ARVs) [5], provided optimism for ending HIV as a public health threat. This progress is now at risk. Funding cuts are disrupting treatment and prevention, increasing morbidity, mortality and infections especially among marginalized groups. Limited access to diagnostics and medicines will worsen treatment quality, inducing resistance to ARVs and medicines for opportunistic infections. These problems do not stay within state borders.

Despite research and programmatic achievements, the transmission of *TB* remains high, due to a combination of insufficient access to treatment, including for Multidrug-Resistant TB (MDR-TB) [6], urbanization and undernutrition [7]. In 2024, USAID was the main source of financial aid, supporting diagnostic, preventive and treatment services, surveillance, research, and the mechanism used by many countries to negotiate prices for TB commodities: the Global Drug Facility (GDF). The collapse of the GDF would cause treatment stockouts and recourse to substandard products, which will fuel transmission and resistance, leading to outbreaks and spread of MDR-TB. While TB programmes in the Global South are at stake, Europe and North America are reaping the rewards of TB research conducted in the Global South [8].

Tremendous efforts to control *malaria*, including the advent of the first two vaccines ever [9], raised hopes of elimination, but control remains elusive due to emerging resistance to treatments, and insecticides [10]; gaps in prevention; and limited access to healthcare. Even if for now most high-income countries remain unaffected, vectors are gradually expanding their geographic distributions due to warming temperatures and altered rainfall patterns, creating risks of reintroduction in countries that achieved elimination [11]. Moreover, the fight against malaria strengthened and expanded health systems, including community health workers and supply chains, as well as vector control measures, which are critical for pandemic prevention, preparedness and response. Downsizing malaria programmes makes countries, regions and the world more vulnerable to (other) public health emergencies of international concern.

## A call to action

The end of US bilateral aid calls for re-prioritisation and enhanced coordination of the global fights against HIV, TB and malaria [12]. Currently, the Global Fund is uniquely positioned to undertake this endeavour, as it financially supports HIV, TB and malaria programmes in most, if not all, countries affected by US spending cuts. This requires a successful replenishment, which seems improbable given the uncertainty about the US position and considering the aid spending cuts announced by other high-income countries. Low- and middle-income countries need to step in, which necessitates an overhaul of the Global Fund governance. We, therefore, propose the following action points.

1. All countries, regardless of income level, should support the current replenishment of the Global Fund.
2. The replenishment mechanism should move towards assessed contributions [13]. Voluntary contributions are unreliable and create an unhealthy power dynamic, as the contributors can threaten to withdraw support. We leave it to governments to decide among themselves what fair contributions for all countries should be, but provide the following reference: 0.01% of the annual gross domestic product (GDP) of all countries combined makes USD 11.5 billion. This can cover the replenishments of the Global Fund (USD 6 billion per year), the WHO (USD 2 billion per year) and Gavi (USD 2 billion per year), and increase the resources of the Pandemic Fund to USD 1.5 billion annually. Agreed and fair assessed contributions may even convince the US to continue its support, if only to avoid becoming a ‘free rider’ in global health security.
3. The Global Fund should commit to overhauling its governance structures. At present, half of the votes on the Global Fund board are reserved for high-income ‘donor’ countries, which is unsuitable for collective action and would become entirely inappropriate when all countries contribute. Should the board continue with only 20 voting members – instead of an assembly in which all countries are represented equally – the votes should be distributed to geographical constituencies representing equal numbers of people. This may take time, but the Global Fund should make this commitment before asking contributions from low- and middle-income countries.
4. The Global Fund should commit to adhere to the Lusaka Agenda, thus making a stronger contribution to primary healthcare; playing a catalytic role towards domestically financed health services; strengthening joint approaches for achieving equity in health outcomes; achieving strategic and operational coherence; and coordinating approaches to products, research and development and regional manufacturing [14].

These four actions would save essential elements of the global responses to HIV, TB and malaria and set a central and collaborative mechanism for global health security on a path towards the principles of Global Public Investment: all benefit, all contribute, all decide [15].
